# CircRNAs in hematopoiesis and hematological malignancies

**DOI:** 10.1038/bcj.2016.81

**Published:** 2016-10-14

**Authors:** A Bonizzato, E Gaffo, G te Kronnie, S Bortoluzzi

**Affiliations:** 1 Department of Women's and Children's Health, University of Padova, Padova, Italy; 2 Department of Molecular Medicine, University of Padova, Padova, Italy

## Abstract

Cell states in hematopoiesis are controlled by master regulators and by complex circuits of a growing family of RNA species impacting cell phenotype maintenance and plasticity. Circular RNAs (circRNAs) are rapidly gaining the status of particularly stable transcriptome members with distinctive qualities. RNA-seq identified thousands of circRNAs with developmental stage- and tissue-specific expression corroborating earlier suggestions that circular isoforms are a natural feature of the cell expression program. CircRNAs are abundantly expressed also in the hematopoietic compartment. There are a number of studies on circRNAs in blood cells, a specific overview is however lacking. In this review we first present current insight in circRNA biogenesis discussing the relevance for hematopoiesis of the highly interleaved processes of splicing and circRNA biogenesis. Regarding molecular functions circRNAs modulate host gene expression, but also compete for binding of microRNAs, RNA-binding proteins or translation initiation and participate in regulatory circuits. We examine circRNA expression in the hematopoietic compartment and in hematologic malignancies and review the recent breakthrough study that identified pathogenic circRNAs derived from leukemia fusion genes. CircRNA high and regulated expression in blood cell types indicate that further studies are warranted to inform the position of these regulators in normal and malignant hematopoiesis.

## Introduction

**Figure 1:**
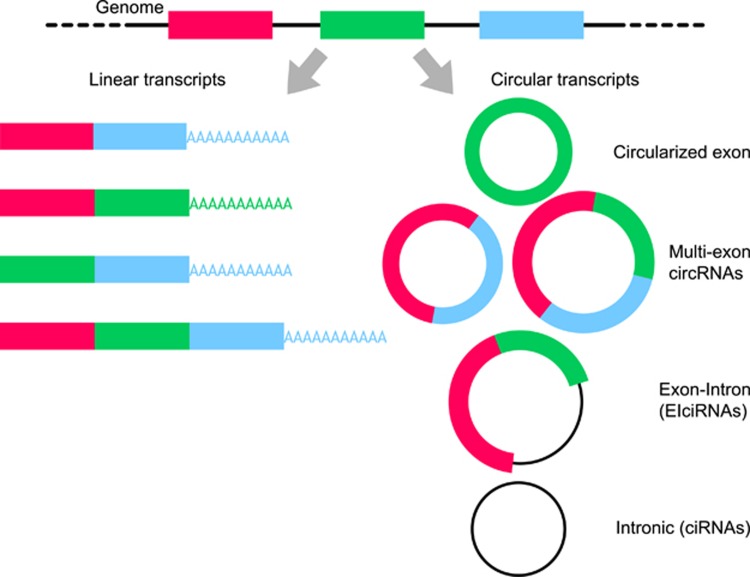
Linear and circRNAs. CircRNAs are produced by backsplicing, and combinations of exons and introns give rise to different products, including single circularized exons, circRNAs formed by two or more exons, by exon and retained intron sequences (EI-ciRNAs) and by intronic sequences only.

Circular RNAs (circRNAs) are covalently closed RNA molecules, in which the 3′- and 5′-ends are linked in a non-collinear way by a process called back-splicing.^1^ Unlike in linear RNA splicing, a splice donor site is joined to a splice acceptor site upstream in the primary transcript, yielding a circRNA.^1^ CircRNAs can be formed by circularization of a single exon, two or more exons,^2^ both exons and intron sequences (exon–intron circRNA, EIcirRNA)^3^ or intronic sequences only (circularized intron RNA; ciRNA)^4^ (Figure 1). Several circular isoforms can be produced from a given gene, and different circRNAs from the same gene may show distinct expression profiles, as reported for *circSTAU2a* and *circSTAU2b*.^5^

Circularity confers specific properties to circRNA: they are highly stable, resistant to RNAse R and have longer half-lives compared with linear RNAs^6, 7^ and tend to accumulate in cells with a low proliferation rate.^8^ Detection of circRNA in human body fluids such as plasma^9^ and saliva^10^ indicates circRNAs as potential disease biomarkers.

The first description of circRNA dates back to several decades ago. Recently, circRNAs were relaunched by RNA-seq-based studies as an RNA species with high relevance for molecular biology and molecular oncology, and today over 10 000 human circRNAs have been identified.^11, 12, 13, 14, 15, 16, 17^

CircRNAs are non-poly-adenylated and coincidental discovery of circRNA in the past can be attributed to RNA extraction methods that mainly used polyA selection. Naturally occurring single-stranded covalently closed RNA molecules were first described in plant viroids^18^ and were valued for their peculiar structure that allows for rolling circle replication.^1^ A few studies in the nineties reported non-canonical splicing with scrambled exons of candidate tumor suppressor gene *DCC*,^19^ ‘missplicing' of *ETS1* transcripts^20^ and murine *Fmn*,^21^ and exon circularization in nuclear extracts.^22^ Moreover, whereas early in development the mRNA of therian *SRY* is translated into the protein that triggers the sex-determining transcriptional cascade, in adults *SRY* transcripts are found as cytoplasmic circular *SRY* (*cSRY*) not particularly bound to polysomes^23^ and later proven to efficiently sponge miR-138.^24^ Other primary RNAs were found to be processed into circRNA isoforms such as MLL (*KMT2A*),^25^ *ETS1*,^26^ *CYP2C18*,^27^ *SLC8A1*^(ref. 28)^ and dystrophin (*DMD*)^29^ transcripts. Examples of circRNAs corresponding to linear noncoding RNA, as well as antisense RNA were also detected.^30, 31^

**Figure 2:**
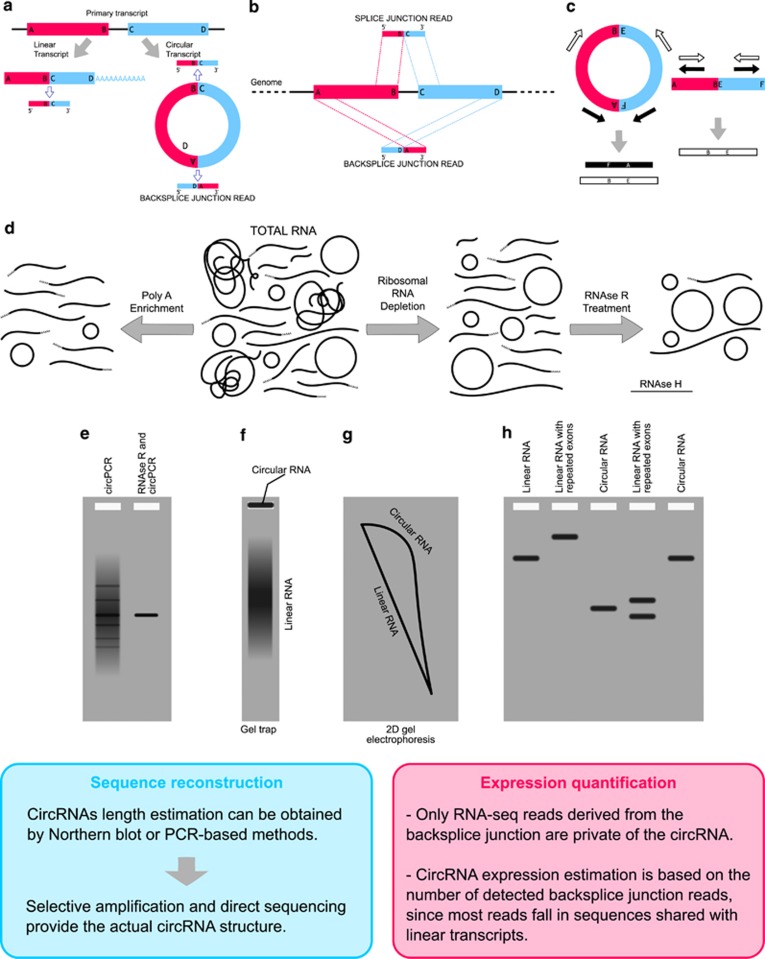
Molecular methods for circRNA detection, validation and study. (**a**) CircRNA detection from RNA-seq data grounds on the identification of sequence reads encompassing the backsplice junction; (**b**) Backsplice reads map to the genome in chiastic order (two segments of a single read align separately in reverse order) due to the backsplicing in circRNAs biogenesis. (**c**) Convergent primers (white arrows) designed in adjacent spliced exons amplify both linear and circular isoforms, whereas primers that are divergent in the linear transcripts (black arrows) can be used to specifically amplify the circular isoform; (**d**) PolyA enrichment protocols deplete circRNAs, whereas ribosome depletion and RNAse R protocols enrich circRNAs; (**e**) RNAse R digestion before reverse transcription–PCR lowers the amount of false-positive amplicons facilitating circRNA validation; (**f**) Gel Trap electrophoresis allows isolate the circular and linear fractions of the input RNA, as circRNAs are hold in the well; (**g**) Two-dimensional acrylamide gel electrophoresis separates the circular RNA fraction in an off-diagonal curve; (**h**) RNA migration in agarose gel before and after a mild RNAse H treatment resulting in a single cut per molecule shows that circular molecules bearing a ‘backsplice' junction are discriminated from linear ones deriving from a duplication event, as only circRNA results in a single band after being cut once (**f**–**h** re-elaborated from^56^).

Most of the above-mentioned studies postulated relevant biological functions for circular RNAs but were only confined to certain genes. In any case, shortly after publication of circular forms of an INK4/ARF-associated non-coding RNA^30^ numerous studies embarked on transcriptome wide circRNA analysis showing developmental stage- and tissue-specific expression, and specific regulatory roles for circRNAs were suggested.^2, 13^ These new data triggered the interests of the scientific community resulting in the development of molecular methods to study circRNAs and of microarray platforms to measure expression levels of circRNAs (Figure 2), as well as the implementation of bioinformatics software to detect and discover circRNAs from RNA-seq data (Table 1) posing the basis for further experimental circRNA characterization, and for circRNA quantification and differential expression testing. Moreover, several available circRNA databases and web resources (Table 2) could be rather useful to explore putative circRNA interactions and functions.

Several studies on circRNAs in blood cell types and hematologic malignancies were recently conducted and will be discussed below.

## CircRNA biogenesis

### Back-splicing

As anticipated, circRNA loops are generated by back-splicing from immature RNA, where ends are joined in a non-collinear way. CircRNAs are derived from Pol II transcripts just like linear transcripts. Back-splicing requires the spliceosomal machinery^32, 33, 34^ as revealed by treatment of HeLa cells with a splice inhibitor followed by nascent RNA purification.

In the majority of cases, the generation of circRNA happens at the expense of their corresponding mRNA isoforms, and is characterized by the usage of canonical splice sites that precisely flank head-to-tail junctions of circular transcripts. In some cases, transcripts of specific genes are predominantly spliced into the circular isoform.^11, 12^ Ashwal-Fluss *et al.*^32^ demonstrated that circularization and splicing of linear forms compete against each other. Kelly *et al.*^35^ confirmed a direct correlation between exon skipping and circularization. Thus, circRNA biogenesis and regulation of mRNA production are tightly linked.

### *Cis* regulatory features

CircRNA-forming exons are often flanked by particularly long introns, possibly reducing splicing efficiency.^12, 16^ Moreover, in humans these long introns are markedly enriched in ALU repeats,^12^ and complementary sequences in introns are involved in specific folding of primary transcripts that favor circularization.^32^ In the *Sry* gene, the activation of an upstream promoter triggers the synthesis of a primary transcript containing inverted repeats needed for circRNA production.^36^ As drosophila RNA circularization does not appear to be driven by structural complementarity of exon-bordering sequences but only determined by the length of exon-flanking introns, inverted repeats alone do not fully explain the production of circRNAs in eukaryotes. In addition, regulation of the dynamic expression of circRNAs in different cell types is likely also dependent on control by *trans*-acting factors.^37^

### *Trans*-acting factors

Besides the role of flanking sequence elements, introns encasing circRNAs are highly enriched in RNA A-to-I editing events.^12^ In fact, knockdown of RNA-editing enzyme ADAR1 upregulated circRNA expression, favoring a mechanism of circRNA biogenesis whereby ADAR1 antagonizes circRNA expression by melting stems of RNA–RNA interactions within introns that putatively promote circularization.^12^

The Muscleblind (MBL) family of splicing factors was also shown to take part in the regulation of circRNA production by binding specific intronic sites flanking circularized exons.^32^ Intriguingly, in the fly circRNA isoform expression of *MBL* itself is regulated by MBL protein. Decrease of circRNA expression after MBL knockdown supports a circRNA-promoting role for MBL proteins.^32^

In addition, RNA-binding protein (RBP) Quaking (QKI) regulates the formation of circular RNAs.^38^ QKI dimers bind to specific bipartite sequences termed QKI response elements that are present in many RNAs, including coding mRNAs and primary miRNAs. Conn *et al.*^38^ investigated the role of QKI in promoting circRNA biogenesis in transforming growth factor-beta-induced epithelial–mesenchymal transition of the epithelial HMLE cell line, demonstrating that the knockdown of the QKI-5 isoform specifically decreases the formation of circRNAs, and that insertion of synthetic QKI response elements in introns mediates circRNA formation. Metabolic tagging of nascent RNAs with 4-thiouridine has been used to study the link between nascent circRNA processing and transcription^17^ showing that the efficacy of circRNA processing from primary transcripts is extremely low. This study also clarified that circRNAs are largely processed post-transcriptionally and confirmed that circRNAs are stable, being thus abundant at a steady-state level and tending to accumulate particularly in cells with low proliferation rates.^8^

### Backsplice as a new form of alternative splicing

Alternative RNA splicing is a complex tightly regulated phenomenon. Since the discovery of split genes robust knowledge was built on splicing prevalence, on complexity of splicing patterns and on molecular mechanisms that determine, regulate or change splicing, including RNA–protein interactions (splicing factors with *in cis* regulatory sites termed silencers or enhancers), RNA–RNA base-pairing interactions involving both *in trans* acting RNAs and *in cis* secondary structure formation, and also chromatin-based effects.^39^

Disease-causing mutations occur in splice sites or in regulatory elements, as well as in genes that encode splicing factors (*U2AF1*, *SRSF2*, *SF3B1* and *ZRSR2*), and there is much interest in developing antisense oligonucleotides to control splicing patterns and using genome editing to correct disease-causing splicing defects. Alternative splicing is highly and commonly deregulated in cancer cells^40, 41, 42^ and specifically impacts prognosis and disease course of myeloid malignancies, including chronic lymphocytic leukemia, acute lymphoblastic leukemia (ALL), acute myeloid leukemia, and myeloproliferative neoplasms.^43, 44, 45, 46^

**Figure 3:**
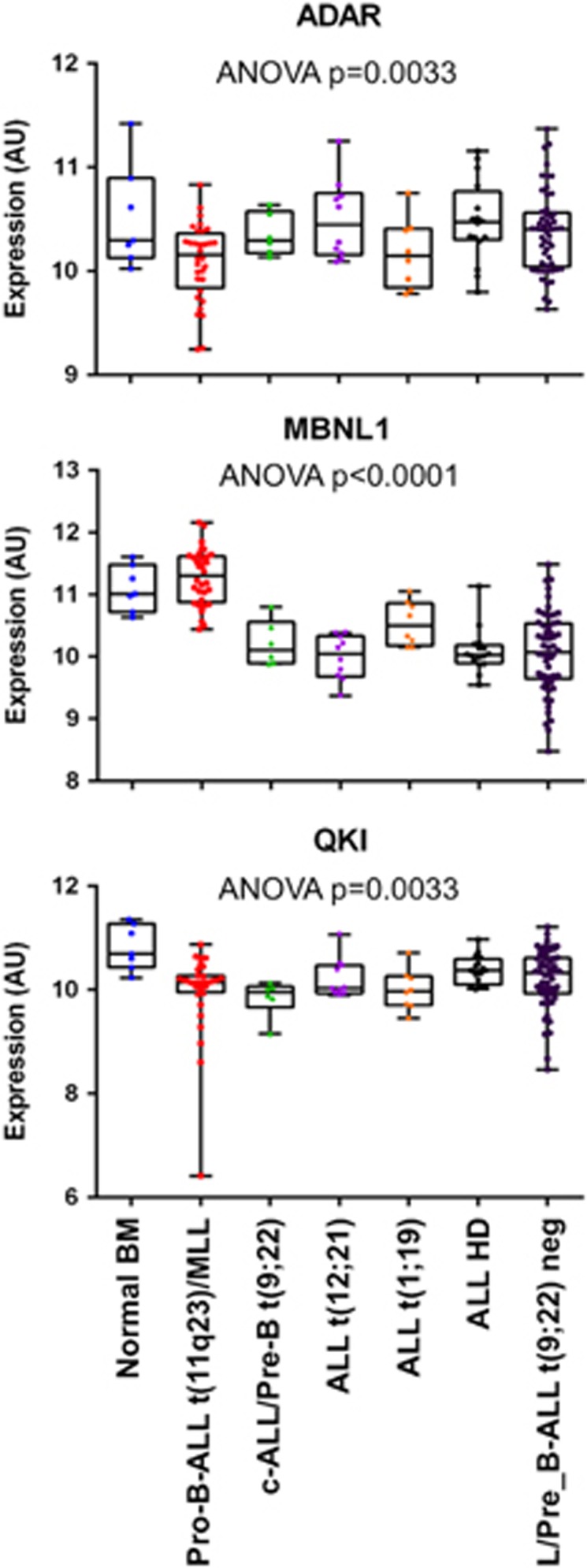
Expression variation of enzymes involved in circRNA expression. Gene expression intensities of ADAR1, MBNL1 and QKI in samples of normal bone marrow and six B-cell leukemia subtypes carrying specific genetic aberrations (according to Haferlach *et al.*^87^); expression data obtained with HG-U133 Plus 2.0 (Affymetrix, Santa Clara, CA, USA).

As circRNA biogenesis and splicing are interleaved processes, it can be hypothesized that mutations of splicing factors and/or alterations of regulatory elements have an impact on circRNA biogenesis. RBP involved in circRNA biogenesis might drive developmental regulation of circRNA formation and show deregulation in disease. Distinct expression levels of ADAR1, MBNL1 and QKI in normal bone marrow compared with B-cell leukemia subtypes (Figure 3) encourage investigations as also subtle expression variations of ADAR1 were shown to be relevant for RNA circularization.^5^

Alternative splicing is a key mechanism through which fundamental processes during hematopoiesis are regulated,^47^ posing the basis to interpret the consequences of genetic variation. Similarly, there is high demand to study circRNA in normal hematopoiesis, to connect biogenic mechanisms with biological functions of circRNAs, to accumulate fundamental knowledge needed to understand disease mechanisms and to inform strategies for therapeutic intervention.

### CircRNA degradation

As circRNAs are endogenous cell products one might ask which endogenous mechanisms cells have to dispose of circRNA. In general, RNA is degraded by the exosome, a multiprotein complex that reminiscently of the proteasome forms a chamber with helicase activity, which unfolds and then degrades RNA. The degradation is prevalently exoribonucleolytic from the 3′-end, but the exosome catalytic subunit RRP44 also has endonuclease activity.^48^

According to available evidences circRNAs are not degraded by treatments (as tobacco acid pyrophosphatase plus terminator 5′-phosphate dependent exonuclease or highly processive 3′- to 5′- exoribonuclease RNAse R digestion) that normally degrade linear RNA with free ends.^31^ Regarding degradation, in general miRNAs can regulate cleavage of circRNAs. The better-characterized path toward degradation of a circRNA is that of *CDR1-as* (circular antisense transcript deriving from cerebellar degeneration-related protein 1 locus) that even presenting multiple miR-7 binding sites is completely resistant to miR-7-mediated degradation and also resistant to miR-769-mediated degradation, whereas the binding of miR-671 to *CDR1-as* directs Ago2-slicer-dependent cleavage.^31^

Undoubtedly, our understanding of the regulation of circRNA turnover and endogenous degradation mechanisms is limited. We can hypothesize that not only the deregulation of circRNA synthesis but also its degradation are biologically relevant. For instance, a lack of cleavage might result in undesired circRNA accumulation.

## CircRNA conservation and molecular functions

### CircRNAs are evolutionarily conserved

CircRNAs were described in many eukaryotes from yeast to humans^49^ and resulted very conserved at the nucleotide level: Memczak *et al.*^13^ analyzed sequence conservation within circRNAs and showed that 223 human circRNAs with conserved circularization in mice were significantly more conserved in the third codon positions than exons not engaged in circular forms. CircRNAs are also depleted of polymorphisms in miRNA-binding sites.^50^ Beyond apparent sequence conservation, both paralogous and orthologous gene pairs have been reported to express circular transcripts: human *HIPK2* and *HIPK3*, as well as murine *Hipk2/3* produce circRNAs.^12^ Also conservation of circRNAs in terms of exonic sequences, bordering intronic sequences, precise backsplice junctions and expression patterns in mammals and to some extent in *Drosophila* has been recently reported.^5^ The above indications of evolutionary preservation point to a central position of circRNAs in core biological processes.

### CircRNAs are seldom translated

**Figure 4:**
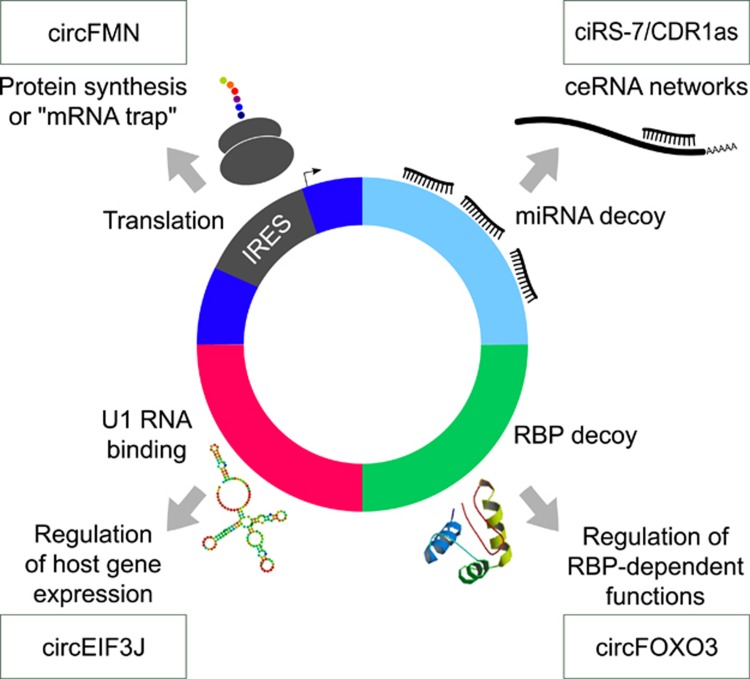
CircRNA functions. Elucidated circRNA functions include the ability to sponge miRNAs thus regulating the silencing of canonical targets (for example, *ciRS-7/CDR1-as* harbors 63 binding sites for miR-7) and participating to ceRNA networks; similarly circRNAs could decoy RBP ultimately regulating the functions in which RBP are implicated (for example, *circ-Foxo3* forms a ternary complex with p21 and CDK2 arresting cell cycle progression); circRNAs can also regulate *in cis* the expression if the gene from which they derive through interactions with the U1 RNA in the U1 RNP in the nucleus (for example, *circEIF3J*); moreover, circRNAs harboring an IRES could be translated to produce peptides or compete with mRNA translation (for example, *circFMN* contains an active translation start site not leading to the protein synthesis).

It has previously been shown that eukaryotic ribosomes can initiate translation on circRNAs containing internal ribosome entry site elements (Figure 4) producing long-repeating polypeptides in the presence of a continuous open reading frame.^51, 52^ Efficient circRNA translation can occur in HEK293^(ref. 34)^ and Hela cells.^53^ Intriguingly, a circRNA can be a sort of ‘*Mobius strip*' with translation generating proteins either recurrently, or variably depending on whether or not the sequence length is a multiple of three nucleotides. A small viroid circRNA directly translated through three completely overlapping open reading frames shifting to a new reading frame at the end of each round has been reported as a natural supercompact ‘nanogenome'.^54^

Even if in principle circRNAs can be translated, the majority of recently discovered and characterized circRNAs seem to have limited coding potential: seldomly associated neither with messenger ribonucleoprotein particles nor with translationally active polyribosomes, suggesting that circRNAs, as a species, are unlikely to be translated into peptides.^55^ The fact that in the same study mass spectrometry failed to identify peptides encoded by backsplice junctions of circRNAs could be due to low sensitivity or to the position of open reading frames outside junctions and does not rule out that part of circRNAs can be translated in some cell types and/or conditions.

A circRNA produced by murine *Fmn*^21^ contains an active translation start site not leading to protein synthesis. In this way, the circular form competes with the linear mRNA both impacting on the linear transcript abundance and providing an ‘mRNA trap' that can sequester proteins of the translation initiation complex. Also, Jeck and Sharpless^56^ uncovered that many single-exon circRNAs contain a translation start site,^57^ further exemplifying this mechanism of protein expression regulation by circRNAs.

### CircRNAs are efficient miRNA sponges and participate to competing endogenous RNA networks

As demonstrated by several studies, circRNAs with multiple miRNA-binding sites are efficient miRNA sponges that participate in the regulation of specific cellular pathways (Figure 4).^24, 58^

*CDR1-as* harbors 63 conserved binding sites for miR-7 displaying high miRNA-binding capacity and miRNA antagonist activity in the brain.^13, 24^ Following this functional description *CDR1-as* was renamed circular transcript *ciRS-7* (circular RNA sponge for miR-7) or called *CDR1-as/ciRS-7*. Notably, the circRNA is completely resistant to miR-7-mediated target destabilization and strongly suppresses miR-7 activity, resulting in increased levels of miR-7 targets, including *EGFR* and *IRS2*.

The previously mentioned *cSRY* decoys miR-138, for which it displays 16 target sites.^12, 24^ Circ-ITCH, a circRNA downregulated in carcinomas, was demonstrated to be a sponge of miR-7, miR-17 and miR-214, by increasing the level of ITCH and ultimately inhibiting the Wnt/β-catenin pathway.^58, 59^ Another circRNA (*hsa_circ_001569*) that acts as a sponge for miR-145 upregulates its targets enhancing cell proliferation and invasion of colorectal cancer.^60^

Recently, specific circRNA–miRNA axes have been shown to regulate cancer-related processes. CircRNAs can have both cancer-promoting and -suppressing roles, depending on the molecular circuits in which they are involved and on the role of the interactors.^58, 59, 61^ CircRNAs can exhibit anticancer effects: as synthetic circular sponges displayed superior anticancer activities compared with the linear sponges, RNA circles open new ways to deliver miRNA sponges with persistent effects.^26^

CircRNAs like linear isoforms can act as competing endogenous RNAs (ceRNAs) that decoy miRNAs and indirectly regulate protein-coding gene expression (Figure 4).^62^ ceRNAs are implied in the progression of cancer and impact on cancer hallmarks.^57^ Being resistant to miRNA-mediated degradation circRNA can presumably also tether RISC components depriving the cellular pool of both miRNAs and RISC effectors.^58^

Following the observations of circRNAs acting as ceRNAs it has been asserted that such a mechanism may be common to all circRNAs. The latter finds support from previously mentioned shortage of polymorphisms in circRNAs' putative miRNA-binding sites. Other studies showed instead that only a minority of expressed circRNAs present multiple binding sites for specific miRNAs, and according to their observations circRNAs are, in general, not bound to miRNA-loaded Argonaute proteins.^56, 63^ In addition, argonaute co-immunoprecipitation experiments did not indicate an appreciable enrichment of circRNA-derived exons among argonaute family-bound transcripts, which would be expected if circRNAs were prevalently acting as ceRNAs. See Thomson *et al.*^64^ for a review on evidences and open questions on endogenous miRNA sponges. According to available data, supported by recent findings^3, 59^ we conclude that some circRNAs can act as ceRNAs, whereas others may be involved in a variety of other molecular mechanisms.

### Interactions with RBPs

The decoy activity of circRNAs could be important also for RBPs (Figure 4). CircRNAs like linear RNAs may interact with RBPs in a sequence-specific and structural motif determined way. CircRNAs could function to store, sort or localize RBPs. Recently, the interaction between *Foxo3* circular RNA and specific proteins was shown to delay cell cycle progression.^65^ Foxo3 is a forkhead box O transcription factor and may behave as a tumour suppressor protein that limits cell proliferation and induces apoptosis and is frequently altered in cancer, shown to be deleted in lymphomas (diffuse large B-cell lymphoma), and translocated with MLL in leukemia.^66^ In healthy cells high *circ-Foxo3* expression was found to be associated with cell cycle progression. Silencing endogenous *circ-Foxo3* promoted cell proliferation, whereas ectopic expression of *circ-Foxo3* repressed cell cycle progression by binding to cell cycle proteins cyclin-dependent kinase 2 (CDK2) and cyclin-dependent kinase inhibitor 1 (or p21). Normally, CDK2 interacts with cyclin A and cyclin E to facilitate cell cycle entry, while p21 inhibits these interactions and arrests cell cycle progression. The formation of the circ-Foxo3–p21–CDK2 ternary complex arrests the function of CDK2 and blocks cell cycle progression. This study identified an oncogenic function of a circRNA and indirectly demonstrated that circRNAs can have distinct functions with respect to that of protein products encoded by the same gene.^65^

### *Cis* regulation of gene expression by circRNAs

Another line of evidence reported *cis*-regulatory roles for specific circRNAs. Exon–intron circRNAs derived from circularization of RNA with intron retention were identified as a subclass of ciRNAs, enriched in the nucleus, associated with Pol II.^3^ Further analyses of two exon–intron circRNAs (*circEIF3J* and *circPAIP2*) showed interactions with Pol II, U1 snRNP and parental gene promoters through sequence complementarity between the U1 snRNA and an U1-binding site, which eventually promote the transcription of the gene from which they derived (host genes), triggering a positive-feedback loop.

Zhang *et al.*^4^ described circular intronic RNAs (ciRNAs) that were found to accumulate in human cells due to a failure in debranching and showed that knockdown of ciRNAs reduced expression of their host genes. One of these abundant RNAs, ci-ankrd52, largely accumulates to its sites of transcription, associates with the elongation Pol II machinery, and acts as a positive regulator of Pol II transcription.^4^ Apparently also non-coding intronic segments of ciRNA transcripts can have a *cis*-regulatory role. *CDR1-as* stabilizes *CDR1* mRNA expression, probably with a sense–antisense-based feedback mechanism, where the antisense circRNA stimulates or stabilizes the sense mRNA with subsequent negative impact on antisense levels.^31^

The fairly well-characterized role of circRNAs to positively modulate host gene expression has foreseeable implications for stability of cell commitment choices such as in hematopoiesis.

In summary, circRNAs can regulate host gene expression but also participate to complex networks in which they compete for the binding of miRNAs (ceRNA networks) of RBPs or even for translation initiation. CircRNAs with different composition in terms of exon–intron inclusion result in a multitude of mechanisms that can affect transcriptome and proteome regulation. Through studies of specific tissues, cell types and conditions the evident versatility of circRNA is expected to reveal insight in all kinds of cellular processes.

## CircRNAs in the hematopoietic compartment

RNA-seq analyses showed dynamic expression of circular isoforms independent of linear transcript dynamics from the same gene^5^ and cell- and differentiation stage-specific expression^2, 13^ prompting lines of research that focused on specific tissues. Knowing that circRNAs interfere in key cellular processes like self-renewal, proliferation and apoptosis there is growing interest to study of circRNA in the hematopoietic compartment. In the hematopoietic tissue, pioneering studies reported circRNA isoforms of key genes such as *MLL*^25, 67^ without receiving much resonance in the research community, probably due to the reported low expression of circular isoforms compared with abundant mRNAs encoding these key transcriptional regulators. In hindsight low expression of circRNAs of the above transcription factor can be understood from recent observations that in specific cell types highly expressed genes (in terms of expressed linear isoforms) give rise to relatively less circRNA compared with moderate- or low-expressed genes.^5^

### CircRNA discovery by RNA-seq in hyperdiploid B-cell precursor-ALL and in sorted normal leukocyte cell populations

Aiming to discover new cancer-specific fusion transcripts in hyperdiploid B-lineage acute lymphoblastic leukemia, Salzman *et al.*^11^ exploited RNA-seq and found many transcripts with permutated exon order, which they called ‘scrambled exons' and attributed to circularized RNAs. In five samples of hyperdiploid B-cell precursor-acute lymphoblastic leukemia they detected hundreds of circRNA transcripts with >700 circular isoforms comprising more than 10% of all transcript isoforms produced from a comparable number of genes. These circRNAs were however not a specific feature of the leukemic cells; PCR verified that scrambled exons were also detected in remission samples of the patients, in HeLa cells and in normal primary human cells. Also in sorted cells populations, naive B cells (CD19+), hematopoietic stem cells (CD34+) and neutrophils, circRNA isoforms expressed by >800 genes were identified, with circRNA expression accounting for >10% of gene expression. This study showed that a particular gene can produce circRNAs in more than one leukocyte type, but single replicate-based preliminary estimations suggest quantitative differences among cell types. This first indication that circRNAs are expressed both in normal and malignant hematopoietic cells informs the number of circRNAs in immature and lineage-specific blood cells but does not provide a more specific and useful interpretation of circRNA relevance for hematopoietic cell functions and pathology. For instance the study reported and validated a few most abundant transcripts with scrambled exons (*ESYT2*, *FBXW4*, *CAMSAP1*, *KIAA0368*, *CLNS1A*, *FAM120A*, *MAP3K1*, *ZKSCAN1*, *MANBA*, *ZBTB46*, *NUP54*, *RARS* and *MGA*) but did not pay particular attention to circRNAs from numerous genes that are important for normal hematopoiesis and present genomic aberrations or deregulated expression in leukemia.

According to published data^11^ we observed that circRNAs from genes related to B-cell differentiation and acute lymphoblastic leukemia (*JAK2*, *PAX5*, *IKZF1*, *ETV6* and *EBF1*) are prevalently present in hyperdiploid leukemia compared with normal leukocytes samples. The latter is likely an underestimation, as only 54 genes were screened and circRNAs supported by only 1 read were not considered.

The same authors also investigated circRNA expression in 15 different cancer and non-cancer cell lines, detecting around 47 000 circle-specific splice junctions from 8500 genes. The validation of 8 candidates confirmed that all were true circRNAs.^2^ The study further specified that highly expressed circRNA showed cell-type-specific increase in expression that was not associated to an increase of the corresponding linear RNA. Notably, among others, the leukemia cell line K562 presented the largest number of genes (16 559) with evidence of circular RNA expression.

A reanalysis of the same data set using the CIRI method^67^ indicated that more universally shared circRNAs tend to have higher expression levels and verified that the expression patterns of linear transcripts of circRNA-encoding exons are more similar in cancer cells compared with non-cancer cell types, whereas cancer cells appear to have more diverse circRNA expression profiles, both considering exonic and intronic circRNAs.

Subsequently, a comparison of CD34+, CD19+, neutrophils and HEK293 (human embryonic kidney cells) considering only a single biological replicate per cell type (with sequencing depth around 20 million reads per samples) was reported.^13^ The study detected 1950 circRNAs of which 939 are exclusively expressed in CD19+ cells, 333 in CD34+, 194 in neutrophils and 60 in HEK293 cells. Nineteen circRNAs resulted to be shared between these cell types. The emphasis of this study was on the demonstration that circRNAs are in part cell-type-specific and are expressed in a developmental stage-related manner.

### CircRNAs in whole-blood samples from healthy individuals

RNA-seq analysis of whole-blood samples^68^ showed that whole blood was very rich in circRNAs, comparable to the cerebellum, with consistent data comparing two biological replicates. Also in this study the emphasis laid on the numeric evaluation and the demonstration that circRNAs are a natural component of the transcriptome. Whole blood is composed of a gamma of different cell types. Moreover, the plasma component may also be a sink for circulating circRNA of non-hematopoietic cell origin and can be in fact explored for disease biomarkers, as previously proposed for solid tumors.^9^ We cannot exclude that plasma samples could also contain exogenous RNA. In malaria infection, thousands of very short circRNAs are produced by *Plasmodium falciparum*,^69^ including dozens of circRNAs harboring >100 binding sites for a given human miRNA, pointing to highly versatile parasite–host interactions. Similarly, as already proposed for virus–host interactions, circRNAs of viral origin might sponge host miRNAs and vice versa.^70^

### CircRNAs in platelets

Alhasan *et al.*^71^ reported circRNA enrichment in platelets that they ascribed mainly to differential decay of linear RNA, considering the particular circRNA resistance to degradation. In the past integration of transcriptome and proteome data of platelets had given somewhat conflicting results.^72^ Extensive degradation of linear RNA isoforms leaving circRNAs intact, which results in an extensive reduction of the translatable RNAs provides now a straightforward explanation for this apparent disparity. This study demonstrated that circRNAs are highly enriched not only in platelets but also in erythrocytes relative to nucleated cells finding that >3000 genes show 17- to 188-fold relative enrichment of circRNAs.

## Fusion-circRNAs derived from chromosomal translocations have oncogenic role

### Fusion-circRNA discovery

**Figure 5:**
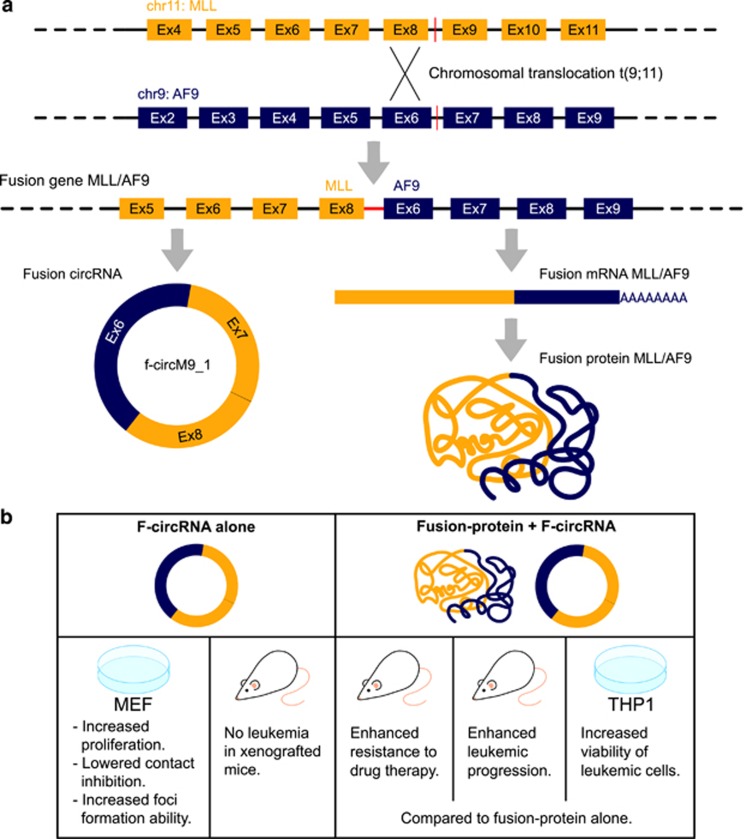
f-circRNAs derived from chromosomal translocations have oncogenic role. (**a**) Transcription of fusion genes generated by cancer-associated chromosomal translocation could generate both linear mRNA coding for oncogenic fusion proteins and f-circRNAs.^73^ The figure depicts the example of *f-CircM9_1* expressed in cells harboring the well-known acute myeloid leukemia MLL/AF9 t(9;11)) translocation: f-CircM9_1 includes two sequences not present in the normal genome, the MLL exon 8 and AF9 exon 6 fusion junction derived from the chromosomal translocation, and the backsplice junction connecting MLL exon 7 with AF9 exon 6; (**b**) f-CircM9 was demonstrated to be proto-oncogenic *in vitro* (increasing proliferation rate and foci forming ability in mouse embryonic fibroblasts, MEF), and required for leukemic cell (THP1) viability. *f-CircM9* alone resulted not sufficient to trigger leukemia *in vivo* when expressed in HSC xenografted in mice. Concurrent expression of f-circM9 and MLL/AF9 fusion protein contributed to leukemia progression *in vivo* and *ex vivo* cells expressing *f-circM9* and MLL/AF9 displayed increased ability to proliferate and to form colonies. Furthermore, *f-circM9* expression in MLL/AF9 mouse model cells increased the resistance to leukemia treatments suggesting that *f-circM9* impacts to pre-clinical therapeutic outcome.

Very recently fusion-circRNAs (f-circRNAs) derived from transcribed exons of chimeric genes generated by cancer-associated chromosomal translocation were discovered and proven to be oncogenic according to *in vitro* and *in vivo* experiments^73^ (Figure 5). Instead of a discovery-driven approach this study used informed guessing to directly detect transcript circularization around the breakpoint/fusion region of two well-known recurrent leukemia-related translocations. The authors hypothesized that juxtaposition of complementary sequences in introns at either side of the fusion regions could favor the formation of circRNAs and searched specifically for circRNAs expressed from fusion genes. f-circRNAs were thus detected by RT–PCR and then confirmed using RNA-seq and custom bioinformatics procedures, in promyelocytic leukemia with a PML/RARα and acute myeloid leukemia with an MLL/AF9 fusion (Figure 5a). Both translocations gave rise to more than one f-circRNA characterized by different backsplice junctions, both in patient samples and in patient-derived cell lines. The discovery was also extended to solid tumors showing f-circRNAs transcription in translocated Ewing sarcoma and lung cancer.

### f-circRNAs are proto-oncogenic and a requisite for leukemic cells viability

Guarnerio *et al.*^73^ showed that f-circRNAs (*f-circPR* and *f-circM9*) expression in leukemic cells increases cell proliferation and clonogenicity and that f-circRNA silencing reverted the phenotype, demonstrating that these f-circRNAs are biologically active and exert pro-proliferative and proto-oncogenic activities(Figure 5b). Moreover, shRNA-based knockdown of *f-circM9* in leukemic THP1 cells resulted in increased apoptosis showing that f-circRNAs have an important role in maintaining the viability of leukemic cells.

### *In vivo* study of f-circRNAs

Human leukemic cells *in vivo* expressing f-circRNAs sustain disease progression in mouse. On the other hand, f-circRNAs alone did not trigger leukemia. Cells expressing *f-circM9* together with the MLL/AF9 fusion protein have an increased ability to proliferate and form colonies than cells expressing the fusion protein alone, strengthening the hypothesis that f-circRNAs contribute to the leukemogenic process *in vivo* (Figure 5b). Human leukemic cells *in vivo* expressing f-circRNAs sustain disease progression in mouse. Moreover, f-circRNAs expression could provide tumor cells protection to standard leukemia treatment, as arsenic trioxide, and confer survival advantage to leukemic cells in response in addition to standard-of-care leukemia treatment with cytarabine. Thus, according to these experiments in a pre-clinical setting *f-circM9* could impact therapeutic outcomes.

Interestingly, Guarnerio *et al.*^73^ argued that the latency of leukemia development in animal models could be due to the absence of f-circRNAs in modeled expression of intron-less fusion genes. Even if it does provide neither data nor hypotheses on the mechanisms underlying the observed pathogenetic effect of f-circRNA, the study^73^ is a breakthrough for leukemia research. In addition, as noticed by the authors, not only the expression of f-circRNA but also the reduction of circRNAs expressed from the non-translocated allele partner genes can contribute to the pathogenic effect.

## Conclusions and outlook

CircRNA expression further challenges a simplistic definition of ‘gene', reinforcing the concept that genes are complex transcriptional units, and that the sequence of a given genomic region is a sort of palimpsest (ancient parchment on which the original text was overwritten multiple times) that contains multiple, interleaved and overlapping information parcels.^74^ Transcripts from the same locus use a common sequence in different ways, and perform distinct biological roles. In addition, circRNAs add new hints to our understanding of the alternative use and reuse of RNA sequences to produce different products and even small RNAs, as known in the case of miRtrons,^75^ of tRNA- or snoRNA-derived miRNAs^74, 76^ and moRNAs,^77, 78, 79^ that are expressed in blood cells,^78, 79^ and were shown to have pathogenic relevance in B-cell lymphomas.^80^ Ultimately gene expression studies need to disentangle the expression of linear and circRNAs expressed from each locus in order to dissect the distinct or complementary processes in which they participate.

A large body of data regarding molecular circuits that control cellular differentiation of the hematopoietic system is available and its deregulation in malignancies in association with genomic lesions is increasingly understood. In hematopoiesis, differentiated cell states are controlled by densely interconnected transcriptional circuits^81^ in a seemingly hierarchical process of binary fate decisions, but the stiffness of cell fate may be more fluid^82^ allowing for epigenetic regulation in response to mature blood cell demand. We envisage that circRNAs studies of hematopoietic cell stages will further elucidate how cell fate fluidity may depend on stably present circRNAs of key cell stage mRNAs.

Gene expression profiling of the protein-coding transcriptome has been very useful for the study of hematopoietic malignancies but will become more complete when integrated with circRNA expression data. Among other things it can be expected to elucidate discordant gene–protein expression often revealed for marker proteins of hematopoietic cell stages (for example, CD10, CD22 and CD38). Further, induced pluripotent stem cell modeling of hematopoietic diseases may also benefit from circRNA studies, hitherto nothing is known about the behavior of cricRNAs in reprogramming procedures of induced pluripotent stem cell generation.

Recent RNA-seq data outlined transcriptional diversity in terms of (linear) alternative isoform-ratio variations among hematopoietic cells^47, 83^ and of non-coding RNA's impact in hematopoietic lineage differentiation.^84^ Specific miRNAs are expressed in a developmental-stage-specific manner.^85, 86^ miRNAs and other small RNAs are differentially expressed in disease.^79^

As in last years we abandoned the concept of the centrality of coding fraction of the transcriptome, the discovery of circRNAs made clear that the study of the linear RNAs only provides an incomplete picture of the cellular complexity. Focusing on linear transcription only we miss important elements, both for data interpretation and experimental design.

Today we have an increased appreciation of circRNA abundance, evolutionary conservation and diversity of functions and interactions. Specific data emerged of high and regulated circRNA expression in normal and malignant blood cells. The recent discovery and functional study of f-circRNAs provided important clues of the oncogenic role of this aberrant circRNA in leukemogenesis and of their relevance in modulating therapeutic outcome. Together these data clearly indicate that further studies of circular isoforms from different cell types and stages of the hematopoietic compartment and by rearranged or mutated genomes are warranted to better estimate the position of these new regulators in hematopoietic cell development and derived malignancies. The route toward elucidating circRNA biology is still long. Even a consistent nomenclature for circRNAs is sorely missed.

Definitely, circRNAs and their diverse molecular interactions participate to the circuitries that regulate the final cellular protein output adding to the richness and complexity of the underlying mechanisms. The particular stability of circRNAs may also make them valuable disease markers that can be identified in various body fluids and we envisage that a better understanding of circRNA biology will inform innovative therapeutic targets.

## Figures and Tables

**Table 1:** CircRNA discovery, characterization and quantification from RNA-seq data

*Name*	*Aligner*	*Map steps*	*Annotation needed*	*Gives gene names*	*Only circRNA*	*Notes*	*References*
CIRI	BWA-Mem	1	No (only for report)	Yes	Yes	Accounts for uncertainty of read mapping to the junction	^67^
Find_circ	Bowtie	2	No	No	Yes		^13^
CIRCexplorer	STAR, TopHat	2	Yes	Yes	Yes		^15^
Testrealign	Segemehl	1	No	No	No	Parses segemehl alignments	^88^
UROBORUS	TopHat, Bowtie	2	No	Yes	Yes	Do not underestimate expression; filters spurious alignments	^89^
NCLscan	BWA, Novoalign, BLAT	3	Yes	Yes	No	>98% precision, test on poly(A)±libraries	^90^
MapSplice	MapSplice	1	Yes	Yes	No	Circular RNA explicit detection from MapSplice v2m (2/2013)	^91^
circRNA_finder	STAR	1	No	No	Yes		^14^
KNIFE	Bowtie	2	Yes	Yes	Yes	Statistical approach to enrich true positives	^92^
PTESFinder	Bowtie	1	No	No	No		^93^

Abbreviation: CircRNA, circular RNA.

Computational methods available for circRNAs prediction. In the past reads mapping to the reference genome with non-collinear exon junctions were often considered artifacts and discarded; with the rise of interest on fusion transcripts produced from rearranged genomes, *trans*-splicing and circRNAs, RNA-seq aligners were improved to consider also these ‘exotic' events, where transcript sequence parts correspond to regions that are remote in the normal reference genome (fusion and *trans*-spliced transcripts) or that map to the genome in chiastic way (that is, two segments of a single read align separately in reverse order due to the backsplicing in circRNA biogenesis; see also Figure 2b). The table presents 10 computational methods that allow blacksplice events identification using different strategies and read aligners; some approaches are specific to predict circRNAs, whereas others were developed with more general purposes, such as read alignment and/or detection of fusion events, and allow also circRNAs detection. For each method, the table indicates the read aligners and number of mapping steps implemented in the prediction strategy, and clarify whether the method requires genome annotations in input, whether it provides an annotation of predicted backsplice junctions in terms of overlapping genes and whether it has been designed explicitly for circRNA identification or is a more general purpose software; the last two columns report additional notes on specific software features and references. Recently, five circRNA prediction tools (circRNA finder, Find_circ, CIRCexplorer, CIRI and MapSplice) have been compared by evaluating the levels of *bona fide* and false-positive circRNAs based on RNase R resistance data, showing that not in all cases the most abundant circRNAs are true positives, that circRNAs identified by a single method only are in general less reliable and that the combination of at least two methods might increase specificity.^94^

**Table 2:** Web resources dedicated to circRNAs

*Web resource*	*Address*	*Main features*	*References*
CircBase	http://www.circbase.org/	Simple circRNA database that provides a searchable table of circRNAs identified by several studies (five on human data) that can be useful to sort newly identified from known circRNAs	^95^
starBase v2.0	http://starbase.sysu.edu.cn/	Focuses on RNA–RNA and protein–RNA interaction networks inferred from CLIP-Seq data sets. Among others, in the miRNA–lncRNAs section, it includes predicted miRNA–circRNA interactions and can be searched to identify known circRNA that potentially sponge a specific miRNA	^96^
Circ2Traits	http://gyanxet-beta.com/circdb/	Useful to explore potential associations of circRNAs with diseases based on predicted interactions of circRNAs with disease-associated miRNAs and on the overlap between disease-associated SNPs to circRNA loci	^70^
CircNet	http://circnet.mbc.nctu.edu.tw/	Provides expression profiles of circRNAs in 464 RNA-seq samples, with circRNA sequences and annotations in term of overlapping genes and interactions	^97^

The table describes four websites that store circRNA expression data and provide useful resources to explore possible circRNA functions.
